# Health-related quality of life 12 years after injury: prevalence and predictors of outcomes in a cohort of injured Māori

**DOI:** 10.1007/s11136-023-03419-9

**Published:** 2023-04-13

**Authors:** Brett Maclennan, Sarah Derrett, Emma Wyeth

**Affiliations:** Division of Health Sciences, Te Roopū Rakahau Hauora Māori a Kāi Tahu (Ngāi Tahu Māori Health Research Unit), University of Otago, PO Box 56, Dunedin, 9054 New Zealand

**Keywords:** Māori, Indigenous, Post-injury outcomes, Predictors, Health-related quality of life, EQ-5D-5L

## Abstract

**Purpose:**

Studies have found that many people who sustain an injury can experience adverse outcomes for a considerable time thereafter. Māori, the Indigenous peoples of Aotearoa me Te Waipounamu (New Zealand; NZ), are no exception. The Prospective Outcomes of Injury Study (POIS) found that almost three-quarters of Māori participants were experiencing at least one of a range of poor outcomes at two years post-injury. The aim of this paper was to estimate the prevalence, and identify predictors, of adverse health-related quality of life (HRQoL) outcomes in the POIS-10 Māori cohort, 12 years after participants sustained an injury.

**Methods:**

Interviewers reached 354 individuals who were eligible to participate in a POIS-10 Māori interview, to be conducted a decade after the last phase of POIS interviews (held 24 months post-injury). The outcomes of interest were responses to each of the five EQ-5D-5L dimensions at 12 years post-injury. Potential predictors (i.e., pre-injury sociodemographic and health measures; injury-related factors) were collected from earlier POIS interviews. Additional injury-related information was collected from administrative datasets proximate to the injury event 12 years prior.

**Results:**

Predictors of 12-year HRQoL outcomes varied by EQ-5D-5L dimension. The most common predictors across dimensions were pre-injury chronic conditions and pre-injury living arrangements.

**Conclusion:**

An approach to rehabilitation where health services proactively enquire about, and consider the broader aspects of, patient health and wellbeing throughout the injury recovery process, and effectively coordinate their patients’ care with other health and social services where necessary, may help improve long-term HRQoL outcomes for injured Māori.

## Introduction

The Prospective Outcomes of Injury Study (POIS) is a cohort study of 2856 New Zealanders aged 18–64 years when injured between 2007 and 2009 [1]. Participants experienced a range of injury types and severities (e.g. 25% were hospitalised for their injury), and POIS obtained extensive information about pre-injury, injury-related and post-injury factors from interviews at 3, 12 and 24 months (on average) post-injury [1, 2]. Data collection for POIS-10 Māori [2], a 12-year follow-up of Māori POIS participants, was completed in July 2021.

A specific aim of POIS has been to provide findings of high relevance and utility to Māori [3]. Like many Indigenous populations, Māori experience significant health and well-being inequities, including inequities in adverse injury outcomes compared to non-Māori [4–6]. Our previous analyses found a substantial proportion of the Māori cohort experienced adverse outcomes to 24 months following their ‘sentinel’ injury (i.e., the injury which led them to being recruited to POIS). Using the EQ-5D-3L [7], 55% experienced pain or discomfort 12 months post-injury, 33% had difficulty performing usual activities, and 21% experienced anxiety or depression [8]. Pre-injury prevalence for these was 10%, 5%, and 6%, respectively. At 24 months post-injury, 19% reported disability (WHODAS ≥ 10) compared to 9% pre-injury [9]. Overall, nearly three-quarters (72%) of Māori participants reported ≥ 1 adverse outcome at the 24-month interview (e.g. poorer health-related quality of life (HRQoL), disability, or not returning to paid employment) [2].

While evidence of adverse injury outcomes persisting well beyond two years is increasing [10], much of it comes from studies focussing on specific populations [11], injury types [12, 13], or injuries categorised as serious [14–22]. A paucity of research with general populations whose injuries vary in type and severity remains. It is vital we understand the longer-term impacts of injuries classed as ‘minor’ as these comprise the majority of injuries, contributing to over two-thirds of years lived with injury-induced disability [23, 24]. Likewise, very little beyond our own work is known about Indigenous injury outcomes, especially long-term. The aim of this paper is to estimate the prevalence, and identify predictors, of problems with HRQoL 12 years post-injury in the POIS-10 Māori cohort.

## Methods

Detailed information on recruitment and data collection for POIS and POIS-10 Māori is available elsewhere [2, 25]. Briefly, individuals from one of five regions of Aotearoa me Te Waipounamu (NZ), aged 18–64 years when sustaining an injury resulting in an Accident Compensation Corporation (ACC) entitlement claim, were invited to participate. ACC is NZ’s universal no-fault injury insurer, and entitlement claimants are those who qualify for earnings-related compensation, rehabilitation costs, or additional support for their injury [26].

Interviews collected socio-demographic, health and wellbeing, and injury-related information from participants up to 12 years post-injury. Injury-related information was also collected from ACC claims data and hospital discharge data from the Ministry of Health’s National Minimum Dataset (NMDS). The NZ Health and Disability Multi-region Ethics Committee granted the study ethical approval (MEC/07/07/093/AM07).

Outcomes of interest were the five dimensions of the EQ-5D-5L [27] (i.e., mobility, self-care, usual activities, pain/discomfort, anxiety/depression), dichotomised (in order to facilitate comparison of our 12-year findings with our earlier HRQoL findings measured using the EQ-5D-3L) into ‘problems’ (slight, moderate, severe or extreme problems) and ‘no problems’. Bivariate analyses tested the association between each outcome and a range of pre-injury sociodemographic, health and sentinel injury-related characteristics (Table 1). Potential predictors of 12-year EQ-5D-5L outcomes were selected based on relevant injury and Māori health literature (e.g. [28–30],) and previous POIS findings [8, 31–33].

**Table 1:** Potential predictor variables and corresponding categories

Variable	Measure	References	Categorisation	References
Pre-injury sociodemographic				
Gender	2006 NZ Census question	[38]	Female, male	
Age at time of injury (years)	2006 NZ Census question	[38]	18–29; 30–44; 45–64	
Occupational group	Categorised using NZ standard classification of occupation	[39]	Professional; technical; trade/manual; unclassified	[40]
Living arrangement	2006 NZ Census question	[38]	Alone; With non-family; With family (immediate/extended);	[41]
Adequacy of household income	Household Economic Survey 2006–07 question	[42]	Adequate (just enough/enough/more than enough); Not adequate (not enough)	[41]
Pre-injury health				
Body mass index (BMI)	Self-reported height & weight		≥ 30; < 30; Undisclosed	[43]
Chronic conditions	NZ Health Survey 2006/07	[44]	0; 1; 2 +	[41]
Depressive-type episode^a^	DSM-III screening questions	[45]	No; yes^b^	[43]
General self-efficacy	Slightly modified version of General Self-Efficacy Scale	[46]	Good (≥ 26); Not good (< 26)	[41]
Hazardous alcohol use^a^	AUDIT-C	[47]	No (men 0–3, women 0–2); Yes (men 4–12, women 3–12);	[43]
HRQoL	EQ-5D-3L dimensions	[7]	No problems; Problems (some/extreme problems)	[41]
Physical activity^c^	New Zealand Physical Activity Questionnaire 2004	[48]	≥ 5 days per week; < 5 days per week	[43]
Prior injury	Affected by prior injury at time of sentinel injury		No; yes	
Recreational drug use^a^			No; yes	[43]
Satisfaction with social relationships			Satisfied (mostly/completely satisfied); Not satisfied (neither satisfied or dissatisfied, mostly/completely dissatisfied)	[43]
Injury-related				
Assault	Injury due to physical assault		No; yes	[41]
Anatomical injury severity	NISS	[35]	NISS 1–3; NISS 4–6; NISS > 6	[43]
Hospitalised	NMDS^d^	[49]	No; yes	[43]
Perceived threat to life			No; yes/maybe	[41]
Perceived threat of severe long-term disability			No; yes/maybe	[41]
Accessing healthcare services			No trouble; trouble/mixed	[41]
Work-related injury			No; yes	[32]

^a^Reference period is the 12 months prior to injury

^b^Participants classified as having a pre-injury depressive-type episode in the year prior to injury if they responded affirmatively to at least one of the three DSM-III screening questions

^c^Reference period is the 7 days prior to injury

^d^Determined using the National Minimum Dataset (NMDS) of hospital discharges. Participants classified as hospitalised if their discharge record listed an external cause of injury code and they had been admitted to hospital or treated for ≥ 3 h at an Emergency Department within 7 days of injury

Bivariate analyses were used to construct a multivariable model for the five outcomes. Potential predictors were included if the p-value from its bivariate test of association was < 0.2. These models were subjected to a stepwise backwards elimination regression analysis [34]. Variables were retained if their associated p-value was < 0.15. Gender, age, injury severity (New Injury Severity Score; NISS) [35], pre-injury EQ-5D-3L dimension status, and days from injury to 12-year interview were forced into each model. Modified Poisson regression was used to estimate the relative risk and confidence interval for each retained variable using robust error variances [36]. Analyses were conducted using Stata/SE 13.1 [37].

## Results

A total of 521 potential participants met the eligibility criteria for POIS-10 Māori. Upon contacting potential participants, 12 were found to have died. This left 509 potential interviewees of whom 354 were successfully contacted and 305 completed an interview.

### HRQoL outcomes 12 years post-injury

One-third (34%) of the cohort were experiencing problems with mobility 12 years post-injury and 20% were having self-care difficulties. Problems performing usual activities were experienced by 30%, and 60% were having problems with pain/discomfort. More than one-quarter (29%) were experiencing anxiety/depression.

### Potential predictors of HRQoL outcomes at 12 years post-injury

Table 2 presents the results of our bivariate analyses. Potential predictor variables included in the multivariable models are those where the resulting *p*-value was < 0.2 (underlined).

**Table 2:** Unadjusted associations between pre-injury sociodemographic, pre-injury health and sentinel injury-related factors and the prevalence of EQ-5D-5L problems at 12-years post-injury (bivariate analyses)

Characteristics		EQ-5D-5L dimension									
		Mobility		Self-care		Usual activities		Pain/Discomfort		Anxiety/Depression	
		*N* = 302		*N* = 302		N = 302		*N* = 302		*N* = 302	
	*N* (%)	*n* (%)	*p*	*n* (%)	*p*	*n* (%)	*p*	*n* (%)	*p*	*n* (%)	*P*
Pre-injury socio-demographic											
Gender											
Female	125 (41)	37 (30)	0.23	25 (20)	0.91	38 (31)	0.95	75 (60)	0.79	37 (30)	0.74
Male	180 (59)	65 (37)		35 (20)		54 (30)		105 (59)		50 (28)	
Age at injury											
18–29 years	86 (28)	16 (19)	< 0.01	7 (8)	< 0.01	14 (16)	< 0.01	41 (48)	0.03	20 (23)	0.41
30–44 years	117 (38)	47 (41)		27 (23)		43 (37)		76 (66)		36 (31)	
45–64 years	102 (33)	39 (39)		26 (26)		35 (35)		63 (63)		31 (31)	
Living arrangements											
Alone	16 (5)	11 (69)	0.01	8 (50)	0.01	9 (56)	0.07	14 (88)	0.06	8 (50)	0.01
Non-family	28 (9)	6 (21)		3 (11)		9 (32)		18 (64)		8 (29)	
Family	257 (85)	85 (33)		49 (19)		74 (29)		148 (58)		70 (28)	
Household income											
Adequate	266 (88)	88 (33)	0.60	47 (18)	0.01	77 (29)	0.16	154 (59)	0.17	69 (26)	0.01
Not adequate	37 (12)	14 (38)		13 (35)		15 (41)		26 (70)		17 (46)	
Occupation											
Professional	91 (30)	28 (31)	0.56	17 (19)	0.15	29 (32)	0.62	47 (52)	0.34	21 (23)	0.20
Technical	63 (21)	19 (30)		6 (10)		15 (24)		38 (60)		21 (33)	
Trade/manual	117 (38)	44 (38)		29 (25)		35 (30)		74 (64)		34 (30)	
Unclassified	14 (5)	3 (21)		3 (21)		6 (43)		7 (50)		2 (14)	
Not working	20 (7)	8 (40)		5 (25)		7 (35)		14 (70)		9 (45)	
Pre-injury health											
Body Mass Index											
< 30	185 (61)	50 (27)	0.01	31 (17)	0.04	51 (28)	0.32	101 (55)	0.05	54 (30)	0.91
≥ 30	104 (34)	46 (45)		28 (27)		37 (36)		71 (69)		29 (28)	
Undisclosed	16 (5)	6 (38)		1 (6)		4 (25)		8 (50)		4 (25)	
Chronic conditions											
None	150 (50)	39 (26)	0.01	21 (14)	< 0.01	39 (26)	0.01	78 (53)	0.01	34 (23)	< 0.01
One	87 (29)	32 (37)		18 (21)		24 (28)		56 (64)		24 (28)	
Two or more	62 (21)	30 (49)		21 (34)		29 (48)		45 (74)		28 (46)	
Depressive episode											
No	210 (69)	67 (32)	0.41	39 (19)	0.48	60 (29)	0.38	118 (57)	0.14	52 (25)	0.03
Yes	94 (31)	35 (37)		21 (22)		32 (34)		62 (66)		35 (37)	
Self-efficacy											
Good	284 (94)	98 (35)	0.22	57 (20)	0.64	88 (31)	0.35	169 (60)	0.52	81 (29)	0.80
Not good	19 (6)	4 (21)		3 (16)		4 (21)		10 (53)		6 (32)	
Hazardous drinking											
No	79 (26)	38 (48)	< 0.01	27 (34)	< 0.01	30 (38)	0.11	49 (62)	0.60	28 (35)	0.15
Yes	223 (74)	64 (29)		33 (15)		62 (28)		129 (59)		59 (27)	
HRQoL (corresponding EQ-5D-3L dimension)											
No problems	*Varies by dimension*	86 (31)	< 0.01	55 (19)	< 0.01	81 (28)	< 0.01	149 (56)	< 0.01	80 (28)	0.34
Problems		16 (67)		5 (83)		11 (65)		30 (88)		7 (39)	
Physical activity											
< 5 days a week	130 (44)	47 (37)	0.37	32 (25)	0.06	45 (35)	0.16	75 (59)	0.82	37 (29)	0.94
5–7 days a week	168 (56)	53 (32)		27 (16)		46 (28)		100 (60)		49 (29)	
Prior injury											
No	235 (77)	70 (30)	0.02	42 (18)	0.13	67 (29)	0.30	128 (55)	< 0.01	65 (28)	0.48
Yes	69 (23)	31 (46)		18 (26)		24 (35)		51 (75)		22 (32)	
Recreational drug use											
No	224 (73)	78 (35)	0.41	47 (21)	0.34	69 (31)	0.70	134 (60)	0.65	58 (26)	0.09
Yes	81 (27)	24 (30)		13 (16)		23 (29)		46 (57)		29 (36)	
Satisfaction with social relationships											
Satisfied	23 (8)	90 (32)	0.04	54 (19)	0.44	82 (29)	0.16	164 (59)	0.31	76 (27)	0.04
Not satisfied	282 (92)	12 (52)		6 (26)		10 (43)		16 (70)		11 (48)	
Injury-related characteristics											
Assault											
No	291 (96)	96 (33)	0.55	58 (20)	0.31	86 (30)	0.38	171 (59)	0.94	82 (28)	0.79
Yes/maybe	12 (4)	5 (42)		1 (8)		5 (42)		7 (58)		3 (25)	
Hospitalised											
No	237 (78)	84 (36)	0.18	51 (22)	0.13	78 (33)	0.05	146 (62)	0.09	72 (31)	0.19
Yes	68 (22)	18 (27)		9 (13)		14 (21)		34 (51)		15 (22)	
Injury severity (NISS)											
1–3	121 (41)	45 (38)	0.24	27 (23)	0.27	43 (36)	0.12	75 (63)	0.73	28 (23)	0.23
4–6	141 (47)	40 (29)		22 (16)		34 (24)		81 (58)		46 (33)	
> 6	36 (12)	14 (39)		9 (25)		12 (33)		21 (58)		11 (31)	
Threat of disability											
No	170 (57)	55 (33)	0.70	35 (21)	0.63	50 (30)	0.82	96 (57)	0.33	42 (25)	0.12
Yes	130 (43)	45 (35)		24 (19)		40 (31)		81 (63)		43 (33)	
Threat to life											
No	268 (89)	88 (33)	0.39	52 (20)	0.76	79 (30)	0.37	156 (59)	0.27	72 (27)	0.11
Yes	33 (11)	13 (41)		7 (22)		12 (38)		22 (69)		13 (41)	
Accessing health services											
No trouble	274 (90)	90 (33)	0.46	55 (20)	0.64	79 (29)	0.11	155 (57)	0.02	79 (29)	0.78
Trouble/Mixed	30 (10)	12 (40)		5 (17)		13 (43)		24 (80)		8 (27)	
Work injury											
No	211 (69)	69 (33)	0.83	42 (20)	0.70	60 (29)	0.43	125 (60)	0.74	57 (27)	0.39
Yes	93 (31)	32 (34)		17 (18)		31 (33)		54 (58)		30 (32)	

### Predictors of HRQoL outcomes at 12 years post-injury

#### Mobility

Four of eight variables retained in the multivariable model were found to have a statistically significant association with mobility problems at 12 years post-injury (Table 3). Those aged 30–44 years when injured were almost twice as likely to be experiencing mobility problems than those aged 18–29 years. The risk of problems was also higher among those who had ≥ 2 chronic conditions compared to those who had none. Participants with a hazardous drinking pattern in the year before injury, and those living with others (family/non-family), were at lower risk of experiencing mobility problems.

**Table 3:** Multivariable analysis of factors associated with EQ-5D-5L problems at 12 years post-injury among the POIS-10 Māori cohort

Factors	EQ-5D-5L dimensions				
	Mobility	Self-care	Usual activities	Pain/discomfort	Anxiety/depression
	N = 285	N = 281	N = 286	N = 289	N = 284
	*RR (95%CI)*	*RR (95%CI)*	*RR (95%CI)*	*RR (95%CI)*	*RR (95%CI)*
Gender^a^					
Female	1.00 *ref*	1.00 *ref*	1.00 *ref*	1.00 *ref*	1.00 *ref*
Male	1.34 (0.97, 1.85)	0.91 (0.54, 1.55)	0.99 (0.71, 1.39)	0.96 (0.81, 1.15)	0.97 (0.66, 1.43)
Age^a^					
18–29 years	1.00 *ref*	1.00 *ref*	1.00 *ref*	1.00 *ref*	1.00 *ref*
30–44 years	**1.89 (1.13, 3.14)**	**2.64 (1.14, 6.11)**	**2.42 (1.35, 4.32)**	1.20 (0.92, 1.55)	1.64 (0.95, 2.83)
45–64 years	1.48 (0.85, 2.55)	1.91 (0.80, 4.60)	**2.13 (1.18, 3.81)**	1.19 (0.91, 1.56)	1.68 (0.94, 3.01)
Pre-injury EQ-5D-3L^a^					
No problems	1.00 *ref*	1.00 *ref*	1.00 *ref*	1.00 *ref*	1.00 *ref*
Problems	1.39 (0.93, 2.07)	**2.43 (1.42, 4.18)**	1.36 (0.87, 2.13)	**1.43 (1.16, 1.75)**	0.79 (0.40, 1.56)
Injury severity^a^					
1–3	1.00 *ref*	1.00 *ref*	1.00 *ref*	1.00 *ref*	1.00 *ref*
4–6	0.92 (0.66, 1.28)	1.04 (0.63, 1.73)	0.81 (0.57, 1.15)	0.99 (0.82, 1.21)	**1.72 (1.16, 2.56)**
> 6	1.27 (0.77, 2.08)	1.83 (0.90, 3.75)	1.24 (0.72, 2.15)	0.99 (0.74, 1.34)	1.67 (0.94, 2.99)
Accessing health services					
No trouble	(Not entered)	(Not entered)	1.00 *ref*	1.00 *ref*	(Not entered)
Trouble/Mixed			1.43 (0.92, 2.22)	**1.49 (1.17, 1.89)**	
Body Mass Index (pre-injury)					
< 30	(Dropped)	(Dropped)	(Not entered)	1.00 *ref*	(Not entered)
≥ 30				**1.28 (1.05, 1.55)**	
Unknown				0.79 (0.45, 1.39)	
Chronic conditions (pre-injury)					
None	1.00 *ref*	1.00 *ref*	1.00 *ref*	(Dropped)	1.00 *ref*
One	1.33 (0.91, 1.95)	1.39 (0.75, 2.57)	1.00 (0.64, 1.56)		1.19 (0.73, 1.91)
Two or more	**1.63 (1.10, 2.40)**	**2.32 (1.30, 4.14)**	**1.56 (1.04, 2.34)**		**2.08 (1.35, 3.20)**
Depressive episode (pre-injury)					
No	(Not entered)	(Not entered)	(Not entered)	(Dropped)	(Dropped)
Yes					
Hazardous drinking (pre-injury)					
No	1.00 *ref*	1.00 *ref*	(Dropped)	(Not entered)	(Dropped)
Yes	**0.61 (0.44, 0.92)**	**0.45 (0.28, 0.72)**			
Hospitalised					
No	1.00 *ref*	1.00 *ref*	1.00 *ref*	1.00 *ref*	1.00 *ref*
Yes	0.66 (0.44, 1.01)	0.60 (0.32, 1.11)	**0.59 (0.36, 0.98)**	0.80 (0.61, 1.05)	**0.54 (0.32, 0.92)**
Household income (pre-injury)					
Not adequate	(Not entered)	(Dropped)	(Dropped)	(Not entered)	1.00 *ref*
Adequate					**0.63 (0.42, 0.93)**
Living arrangements (pre-injury)					
Alone	1.00 *ref*	1.00 *ref*	1.00 *ref*	1.00 *ref*	(Dropped)
With non-family	**0.34 (0.14, 0.81)**	**0.26 (0.07, 0.93)**	0.65 (0.35, 1.19)	0.85 (0.58, 1.25)	
With family	**0.55 (0.33, 0.92)**	**0.35 (0.15, 0.80)**	**0.58 (0.38, 0.89)**	**0.69 (0.54, 0.88)**	
Occupation					
Professional	(Not entered)	1.00 *ref*	(Not entered)	(Not entered)	(Not entered)
Technical		0.44 (0.18, 1.06)			
Trade/manual		1.44 (0.81, 2.57)			
Unclassified		1.45 (0.73, 2.87)			
Not working		0.73 (0.33, 1.60)			
Physical activity					
< 5 days a week		1.00 *ref*	(Dropped)	(Not entered)	(Not entered)
5–7 days a week		**0.62 (0.40, 0.97)**			
Prior injury					
No	(Dropped)	(Dropped)	(Not entered)	1.00 *ref*	(Not entered)
Yes				1.21 (0.99, 1.47)	
Recreational drug use					
No	(Not entered)	(Not entered)	(Not entered)	(Not entered)	1.00 *ref*
Yes					1.31 (0.88, 1.93)
Satisfaction with social relationships					
Not satisfied	(Dropped)	(Not entered)	(Dropped)	(Not entered)	1.00 *ref*
Satisfied					**0.56 (0.33, 0.94)**
Threat to life					
No	(Not entered)	(Not entered)	(Not entered)	(Not entered)	1.00 *ref*
Yes					**1.68 (1.02, 2.77)**
Threat of disability					
No	(Not entered)	(Not entered)	(Not entered)	(Not entered)	(Dropped)
Yes					

Bold indicates characteristics where the p-value of the relative risk analysis was < 0.05 (i.e., the difference in the risk of experiencing problems with the HRQoL outcome relative to the reference group was statistically significant at the 5% level)

#### Self-care

Ten variables were retained in the multivariable model for self-care; six were associated with the outcome (Table 3). Those aged 30–44 years when injured were at greater risk of self-care problems 12 years post-injury than those aged 18–29 years. Also at greater risk were those with self-care problems pre-injury and those with ≥ 2 chronic conditions compared to those with none. Participants with a hazardous drinking pattern, those living with others when injured, and those engaging in moderate to vigorous physical activity ≥ 5 days a week prior to their sentinel injury, were at lower risk of self-care problems.

#### Usual activities

Four of eight variables retained in the usual activities multivariable model had a statistically significant association with the outcome (Table 3). Participants aged ≥ 30 years at the time of their injury were more likely to experience problems 12 years post-injury than those who were younger. Those with ≥ 2 chronic conditions were also more likely to be having problems than those with no chronic conditions. At lower risk of problems were those living with family members at the time of their injury compared to those living alone, and those who were hospitalised for their injury compared to those who were not.

#### Pain/discomfort

Four of nine retained variables had a statistically significant association with pain/discomfort at 12 years post-injury (Table 3). Those experiencing pain/discomfort prior to their injury were 1.4 times more likely than those who were not to be experiencing pain/discomfort at 12 years. At higher risk also were those who had trouble accessing health services for their injury compared to those who did not, and those with a BMI ≥ 30 compared to those with a lower BMI. Those living with their family when injured were less likely than those living alone to be having problems with pain/discomfort at 12 years.

#### Anxiety/depression

Ten variables were retained in the multivariable model for anxiety/depression; six had a statistically significant association with the outcome (Table 3). Participants whose injury was of moderate severity were at 1.7 times higher risk of anxiety/depression 12 years post-injury than those whose injury was of low severity, and those with ≥ 2 chronic conditions were around twice as likely to be experiencing anxiety/depression than individuals with only one (RR: 1.75; 95% CI 1.13, 2.71) or no chronic conditions. Individuals who perceived their injury to be a threat to their life were more likely to be experiencing anxiety/depression 12 year later than those who did not, while those hospitalised for injury were at lower risk than those not hospitalised. At lower risk of anxiety/depression were participants whose pre-injury household income was deemed adequate and those satisfied with their social relationships pre-injury.

## Discussion

The prevalence of HRQoL problems 12 years post-injury in the POIS-10 Māori cohort ranged from 20% for self-care to 60% for pain/discomfort. Estimates were lower than at 3 months post-injury (except for anxiety/depression) [31] but higher than the 12-month estimates (except for usual activities) [8]. Increases from 12 months post-injury are likely to be due, at least in part, to the increasing age of the cohort. Compared to the NZ population aged 25 and over, the POIS-10 Māori cohort had a slightly higher prevalence of mobility problems (30% v. 34%), greater problems with self-care (9% v. 20%), the same prevalence of problems with usual activities (both 30%), slightly lower prevalence of pain/discomfort (63% v. 60%), and a lower prevalence of anxiety/depression (44% v. 29%) [50]. Population norms specifically for Māori are not yet available.

The most common predictors for 12-year HRQoL outcomes were pre-injury chronic conditions (mobility, self-care, usual activities and anxiety/depression) and living arrangements (mobility, self-care, usual activities, pain/discomfort). Individuals who had ≥ 2 chronic conditions at the time of their injury were at greater risk of problems than those with none. This was the case for only usual activities and anxiety/depression at 12-months post-injury [51]. It may be that the burden of chronic conditions is now resulting in problems with mobility and self-care also, either through affecting recovery or simply via the cumulative impact of these conditions over time. Living arrangements, on the other hand, did not predict any HRQoL problems at 12 months post-injury [51] whereas those living with other people, in particular family members, prior to their injury were at lower risk of HRQoL problems at 12 years. It could be that this is a marker for the strength of peoples’ support networks and that those with greater support are less likely to encounter HRQoL problems, as has been found elsewhere (e.g., [52]).

Satisfaction with social relationships pre-injury protected against the only 12-year outcome that living situation did not, i.e., anxiety/depression. This may reflect individuals’ feelings of isolation as much as support in overcoming any long-term consequences of their POIS injury. Previous research has found perceived loneliness [53] and social interaction frequency [54, 55] to be associated with mental wellbeing and depression in older adults. At 12 months post-injury, pre-injury satisfaction with social relationships only protected against problems with self-care. We suggested these support networks may help individuals recover from injury but advised treating the finding with caution given there were only a small number of participants experiencing problems with self-care at 12 months [51]. It is also possible, however, that attrition at 12 years is the reason why satisfaction with social relationships did not protect against problems with self-care.

Another variable that predicted problems with self-care at 12 months, but not 12 years, was pre-injury BMI. It instead predicted problems with pain/discomfort. Those with a pre-injury BMI ≥ 30 were at greater risk of pain/discomfort than those with a BMI < 30. While we recommended treating findings regarding 12-month predictors of self-care problems with caution [51], BMI also predicted 12-month problems with self-care in the total cohort [56]. Previous studies have found an association between obesity and pain [57, 58] and future problems with pain [59–61]. Evidence also suggests that obesity increases the risk of injury [62, 63], particular patterns of injury [64], and adverse injury outcomes [62, 65]. The greater risk of pain/discomfort at 12 years in those with a higher pre-injury BMI may be due to the impacts of obesity, either alone or in exacerbating pain/discomfort associated with the sentinel injury.

Adequacy of household income, on the other hand, no longer predicted problems with pain/discomfort at 12 years after being found to protect against it at 12 months. We surmised that adequate household income may have afforded this group better access to medicinal treatments that relieved pain/discomfort. Having adequate household income pre-injury protected against anxiety/depression at 12 years post-injury. While it is possible that more disposable income has enabled better access to support and services, it is perhaps more likely that the observed changes are due to the association between financial security and anxiety/depression [66]. This relationship may have become more pronounced as the cohort has aged towards retirement. It is less clear why pre-injury income adequacy no longer protects against pain/discomfort.

Those aged 18–29 years at the time of their injury were less likely to be experiencing problems with mobility, self-care and usual activities at 12 years than those who were older. Notably, those aged 30–44 years when injured tended to have higher risk estimates than those aged 45–64 years. This may be an artefact of having missing information for those in the oldest age group who had relatively poorer health. They could have been lost-to-follow-up at a greater rate than those in the oldest age group with relatively better health due to having passed away [67] or because they were too unwell for interview, thereby biasing the relative risk estimates downwards. The highest risk of HRQoL problems at 12 months was in the 45–64 year-old age group, although the only statistically significant association was with mobility [51].

Gender predicted problems with usual activities, pain/discomfort and anxiety/depression at 12 months but not at 12 years. We could not think of a reason why females were at higher risk of problems at 12 months post-injury nor is it clear why females are no longer at higher risk, but attrition is one possibility if less healthy females were more likely to be lost-to-follow-up at 12 years. Another potential explanation is reporting bias if males were more likely to downplay HRQoL problems at 12 months post-injury compared to females. Perceiving one’s injury as a threat of longer-term disability at the time of the sentinel injury event also failed to predict any 12-year outcomes after predicting problems with mobility, usual activities and pain/discomfort at 12 months (and being retained in the models for self-care and anxiety/depression). This could be due to the impact of participants’ sentinel injuries now being less pronounced and no longer significantly impacting on HRQoL outcomes.

One new predictor at 12 years was perceived threat to life; those who felt their injury was a threat to their life at the time of the sentinel injury event were at higher risk of anxiety/depression. The emotional impact of injury may have been greater and longer lasting among this group than in those who only perceived a threat of disability. This is consistent with injury severity, as measured by NISS, which also predicted problems with anxiety/depression at 12 years after predicting no outcome at 12 months. These findings are partially supported by previous studies, although comparisons are difficult given the dearth of research examining injury outcomes beyond two years. Brasel et al. (2010) [68] found hospitalised patients’ perceived injury severity to predict mental health status six months later—greater perceived injury severity was associated with poorer mental quality of life. Perceived severity was not associated with clinical severity scores [68] which have been found to have no association with psychological distress up to 24 months post-injury [69, 70].

Those experiencing difficulties with mobility, self-care or anxiety/depression prior to their injury were more likely to be experiencing difficulties with the corresponding outcome at 12 months. However, the only statistically significant association was between pre-injury problems with self-care and 12-month problems with self-care. Conversely, pre-injury pain/discomfort predicted pain/discomfort at 12 years after having no association with pain/discomfort at 12 months. Reasons for these differences are unclear. Mobility problems and anxiety/depression due to the cohorts’ increasing age and/or other significant events post-injury may have disproportionately increased in the group who had no pre-injury problems with these outcomes. Alternatively, those who had problems with these outcomes pre-injury may have actively sought treatment for them over the past 12 years or otherwise reached a point where they no longer consider these a problem. These scenarios would diminish the relative risk of these problems at 12 years between the two groups (i.e., those with and those without these problems pre-injury), and may have occurred to a greater extent for mobility and anxiety/depression than for self-care. Indeed, evidence shows mobility problems are associated with anxiety/depression [71]. Combined with lower statistical power at 12 years relative to the 12-month analyses, this may explain why pre-injury mobility and anxiety/depression problems were not statistically significant predictors at 12 years.

The 12-year finding for pre-injury pain/discomfort seemingly contradicts the finding for pre-injury mobility. An intuitive reason for increasing mobility problems is increased pain/discomfort but several factors can lead to mobility problems, including neurological and cardiovascular conditions [71]. The sentinel injury may have been the cause for much pain/discomfort throughout the cohort at 12 months such that pre-injury pain/discomfort was not a predominant factor in the outcome. Pre-injury pain/discomfort could be a predictor of the outcome at 12 years because much of the pain/discomfort attributable to the sentinel injury may have diminished. Pre-injury pain/discomfort could be a marker for those with long-term chronic pain or those more sensitive to pain [72].

Occupation (self-care), prior injury (pain/discomfort) and recreational drug use (anxiety/depression) were retained in the 12-year multivariable models for the same outcomes they predicted at 12 months but were no longer statistically significant. Potential reasons for this include attrition and lower statistical power for 12-year analyses compared to 12-month analyses.

New predictors at 12 years post-injury were physical activity, hospitalisation, and drinking behaviour. Participants who were exercising 5–7 days a week prior to injury were less likely that those exercising < 5 days to experience difficulties with self-care. The benefits of regular exercise on physical and mental health are well established [73], and evidence suggests it can have a positive effect on health outcomes long-term [74]. Perhaps our finding reflects this, and, in future, physical exercise will show protection against problems with the other HRQoL outcomes as well.

Those hospitalised because of their sentinel injury were less likely to be experiencing problems with usual activities and anxiety/depression. Hospitalised participants possibly received better rehabilitation and care compared to those not hospitalised. This is consistent with our finding for those who had trouble accessing health services for their injury. They were at higher risk of pain/discomfort at 12 years post-injury, perhaps due to insufficient treatment for their injury. Unlike at 12 months, trouble accessing health services did not predict problems with usual activities and anxiety/depression at 12 years. Perhaps those who did have trouble accessing health services for their injury have since recovered to the point where they are no longer emotionally affected by their injury and have been able to resume their usual activities (or at least what they now define as their usual activities) but still experience pain/discomfort that has largely alleviated among those who did not have trouble.

Pre-injury drinking behaviour, like hospitalisation, also had a protective effect for two outcomes. Those classified as hazardous drinkers (i.e., consuming alcohol in a pattern that “increases the risk of harmful consequences for the user or others” [75]) were less likely to be experiencing problems with mobility and self-care at 12 years than those who were not. We cannot think of any plausible reason why hazardous drinking would have a protective effect on these outcomes. The AUDIT-C (range 0–12) [47] was used to measure drinking behaviour. Females who scored ≥ 3 and males who scored ≥ 4 were classified as hazardous drinkers. Studies have found variation in optimal AUDIT-C cut-off points across different populations [76] and it is possible ours were too low.

The finding of a protective effect is somewhat consistent with the “J-shaped alcohol-health curve” observed in several studies examining alcohol consumption and health outcomes [77]. Perhaps we would have observed something similar had we used more than two categories of hazardous drinking (e.g., no/low, moderate, high). Even so, more recent epidemiological evidence shows that the J-shaped alcohol-health curve is highly likely to be an artefact of study design. Purported health benefits of low to moderate alcohol consumption are increasingly doubtful, and it is more likely that alcohol consumption is a marker of better health rather than a cause of it [77, 78]. That may be what we are observing here, and although we adjusted for pre-injury chronic conditions and HRQoL status, hazardous drinking may be capturing another dimension of health. Drinking behaviour can also vary throughout life [79] and we do not know how subsequent drinking behaviour in the intervening years may be impacting on 12-year outcomes.

This is one of only a small number of studies to have examined HRQoL outcomes beyond two years in a general population cohort of injured individuals. Even fewer studies have focussed on a cohort whose injuries varied in type and severity and where most were not hospitalised because of their injury. It is the only study we are aware of that has examined long-term HRQoL outcomes specifically in an injured Indigenous cohort and it is a strength that we have been able to follow-up over half of the sizeable Māori cohort to 12 years post-injury. Nonetheless, our analyses and the statistical precision of our estimates have been restricted by relatively limited statistical power. Consequently, there may be predictors of 12-year HRQoL outcomes that our analyses have not revealed. We used the scientific literature, including our own previous findings, to select variables for our analyses and to inform the regression models. This was done in an effort to overcome the limitations of stepwise regression [80], ensuring our final models were not simply produced by subjecting numerous variables to this method and reducing the chance of extraneous variables being identified as predictors.

The potential for attrition to be influencing our findings is considerable, at least in comparison to our 12-month findings [51, 81]. This may be biasing our observed estimates and limiting the generalisability of our findings. An analysis of non-participation at 12 years post-injury would provide more insight into this. Other potential limitations are likely to be having little impact on our findings. Considerable effort was made to address response error by reassuring participants that taking part in the study would not affect their healthcare or injury compensation in any way. It was highlighted that POIS was being conducted independent of ACC and no individual data was being shared with the organisation. Earlier POIS analyses suggest that any bias from recalled pre-injury health information, collected from participants three months (on average) after their injury, is also likely to be minimal [82].

We made a deliberate decision to focus on pre-injury and injury-related predictors to inform policy, decision-making and practice early on in people’s recovery pathway that may prevent long-term HRQoL problems. A limitation of this is that we do not know what influence behaviour and events in the years since is having on 12-year outcomes. POIS-10 Māori has collected information on major life events, changes in employment, and changes in health in the decade since the 24-month post-injury interview [2]. Findings from this study will help inform future POIS analyses utilising longitudinal data that examine the impact of post-injury factors on 12-year outcomes.

Our findings support a proactive, holistic, integrated approach to the rehabilitation of entitlement claimants. Claimants’ overall health and wellbeing, including comorbidities, living arrangements and social supports should be considered as part of their injury treatment plan. This could, for example, include a focus on the benefits of developing and sustaining a routine of regular physical activity and, if necessary, reducing one’s BMI to improve future HRQoL outcomes. Attention should be paid to an individual’s perceived severity of their injury, particularly in relation to their mental wellbeing. There may well be benefit in proactively following-up with injured patients, particularly those not hospitalised for their injury and/or who are living alone, to see how their recovery is progressing, how they are coping, directing them to any support they may be eligible for and would likely benefit from, and providing advice on further actions they could take to optimise their recovery and HRQoL. Having a process to ensure effective coordination for claimants between the necessary health and social services to optimise their HRQoL outcomes would be particularly useful. Findings from previous research (e.g., [28, 83]) highlight the importance of culturally safe health services for Māori that are easily accessible in order to facilitate obtaining appropriate treatment for injury and other health and wellbeing concerns.

## Data Availability

The study data cannot be shared due to ethical constraints.

## Code availability

Code for the statistical analyses is available from the corresponding author upon reasonable request.
